# What is your diagnosis? Keloidal cord-like lesion on the leg^☆^^☆☆^

**DOI:** 10.1016/j.abd.2019.07.008

**Published:** 2020-03-19

**Authors:** Flaviano da Silva Oliveira, Nadya Picanço Lopes, Carolina Talhari, Antonio Schettini

**Affiliations:** a Tropical Dermatology Clinic, Fundação Alfredo da Matta de Dermatologia e Venereologia, Manaus, AM, Brazil; b Department of Dermatopathology, Fundação Alfredo da Matta de Dermatologia e Venereologia, Manaus, AM, Brazil

**Keywords:** Histology, Lacazia, Lobomycosis

## Abstract

We report a 74-year-old male presented to an outpatient dermatology clinic in Manaus, Amazonas, with a one-year history of pruritic, keloidal lesions on his left lower extremity. Histopathology showed round structures in reticular dermis. Grocott methenamine silver stain revealed numerous round yeasts with thick double walls, occurring singly or in chains connected by tubular projections. The diagnosis was lobomycosis. Although the keloidal lesions presented by this patient are typical of lobomycosis, their linear distribution along the left lower limb is unusual.

## Case report

**Figure 1:**
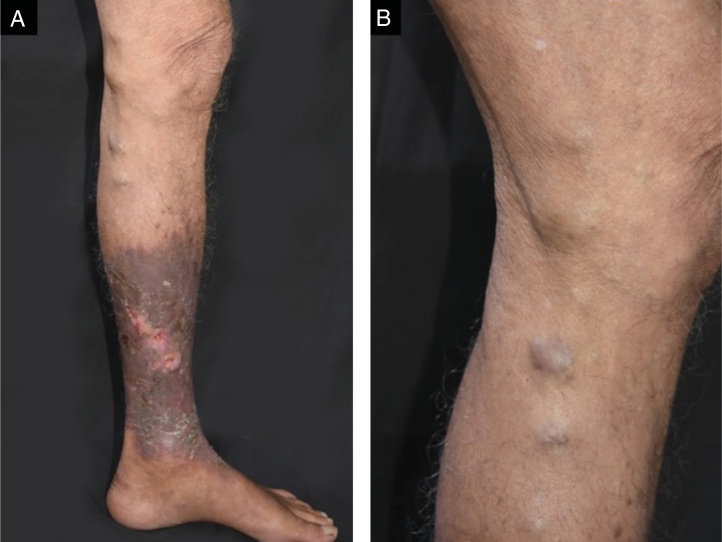
Ulcers with perilesional hardening of the skin, erythema and desquamation were seen on the left leg (A); cord-like, hard, brownish nodular lesions were also observed along the medial aspect of his left thigh and leg at physical examination (B).

In 2005, a 74-year-old male presented with a one-year history of pruritic, cutaneous lesions on his left lower extremity. Physical examination revealed an erythematous plaque and cord-like nodular lesions on the left thigh and leg. Culture, histopathological and mycological examinations were performed at that time but the patient was subsequently lost to follow-up until 2017, when he returned with complaints of recurrent, secondary bacterial infections on the left lower extremity. On physical exam, ulcers with perilesional induration, erythema, and desquamation were noted on the left leg (Fig. 1A). Additionally, cord-like, firm, brown, nodules were observed along the medial aspect of his left thigh and leg (Fig. 1B). Routine labs were unremarkable.

What is your diagnosis?

a) Lymphangitic cutaneous leishmaniasis;
b) Nontuberculous mycobacteriosis;
c) Lobomycosis;
d) Sporothricosis.

**Figure 2:**
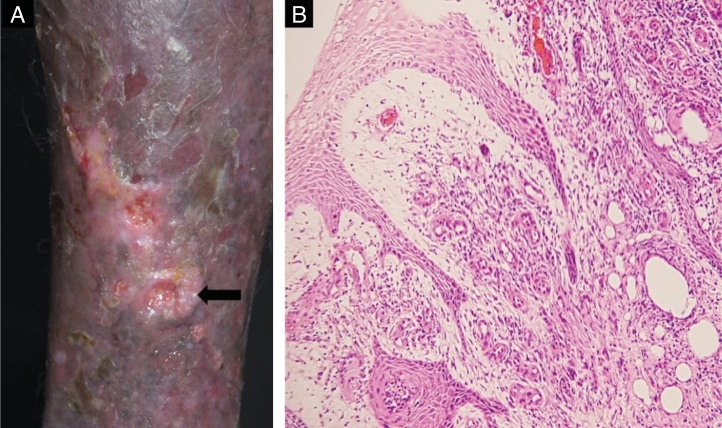
(A) Biopsy site (arrow). (B) Histopathology demonstrated hyperkeratosis and acanhosis, collagen fibroplasia vascular neoformation and diffuse inflammatory infiltrate consisting of lymphocytes, epithelioid cells, giant cells and hemosiderin-laden histiocytes were present in the papilar dermis (Hematoxylin & eosin, ×100).

**Figure 3:**
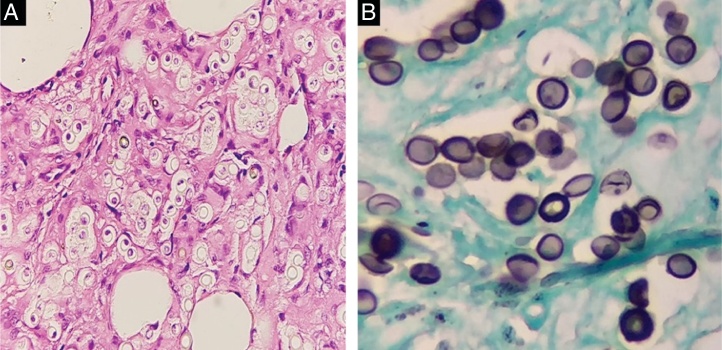
(A) Round structures were seen in reticular dermis (Hematoxylin & eosin, ×200). (B) Grocott's methenamine silver stain revealed numerous round yeasts arranged containing thick double walls, occurring singly or in chains connected by tubular projections (Groccott, ×400).

Cutaneous biopsy was performed on the ulcer border (Fig. 2A). Histopathology showedy shows hyperkeratosis and epidermal acanthosis with a diffuse, predominantly lymphohistiocytic infiltrate. Giant cells, hemosiderin-laden histiocytes and increased vascularity are noted in the papillary dermis (Fig. 2B). Round structures are present in reticular dermis (Fig. 3A), and Grocott's methenamine silver stain reveals numerous round yeasts with thick double walls, occurring singly or in chains connected by tubular projections (Fig. 3B).

Diagnosis: Lobomycosis.

## Discussion

Lobomycosis, also known as Jorge Lobo's disease and lacaziosis, is a subcutaneous, chronic, granulomatous mycosis initially described by Jorge Lobo, in 1931. There are no systemic manifestations in lobomycosis.^1^, ^2^ Due to the similarity of its etiological agent, *Lacazia loboi*, to *Paracoccidioides braziliensis*, the mycosis had also been described as keloidal blastomycosis.^3^ *Lacazia loboi* has never been isolated in culture from humans or animals. Since the initial description of lobomycosis in patients from the Brazilian Amazon Region, other cases have been reported in tropical and subtropical regions of Latin American countries.^2^, ^4^ The few lobomycosis reports from outside these regions have occurred predominantly in patients who had traveled through endemic areas. In 2008, cases were diagnosed in South Africa in patients with no history of travelling to known endemic areas.^5^

An important fact related to this mycosis was the discovery, in the Florida coast in 1970, of a dolphin with a clinical and histopathological disease similar to human lobomycosis.^2^, ^6^ Subsequently, other dolphins with the same disease were found in the Atlantic coast of the United States, coastal region of Latin American and Caribbean countries, coastal region of the Southern Brazilian states of Rio Grande do Sul and Santa Catarina, Pacific coast and Indian Ocean.^7^ There is only one documented case of dolphin to human transmission of lobomycosis: an aquarium attendant who worked in an aquarium pool with an infected bottlenose dolphin (*Tursiops truncatus*) caught in Bay of Biscay, Spain.^8^

The disease is clinically characterized by the presence of nodular, verrucous or keloidal lesions, which are found alone or in plaques, localized or disseminated, with a generally long evolution. The most common clinical presentation is keloidal, characterized by firm, shiny, pinkish-brown to brown nodules.^2^ Ulceration may occur in areas subject to trauma. Development of squamous cell carcinoma in long-standing lesions may occur.^9^

Another clinical aspect that drew attention in this patient was the linear arrangement of lesions along the leg and thigh. It is admitted that the spread of the disease occurs by contiguity, and through lymphatic route; however, an aspect similar to that observed in the present case is not common.

## Financial support

None declared.

## Authors’ contributions

Flaviano da Silva Oliveira: Intellectual participation in the propaedeutic and/or therapeutic conduct of the studied cases.

Nadya Picanço Lopes: Obtaining, analysis, and interpretation of the data; intellectual participation in the propaedeutic and/or therapeutic conduct of the studied cases.

Carolina Talhari: Approval of the final version of the manuscript; conception and planning of the study; elaboration and writing of the manuscript; obtaining, analysis, and interpretation of the data; effective participation in research orientation; intellectual participation in the propaedeutic and/or therapeutic conduct of the studied cases; critical review of the literature; critical review of the manuscript.

Antonio Schettini: Approval of the final version of the manuscript; conception and planning of the study; elaboration and writing of the manuscript; intellectual participation in the propaedeutic and/or therapeutic conduct of the studied cases; critical review of the literature; critical review of the manuscript.

## Conflicts of interest

None declared.
